# Paving the path to the future of carbogenic nanodots

**DOI:** 10.1038/s41467-019-10394-8

**Published:** 2019-06-03

**Authors:** Navneet C. Verma, Aditya Yadav, Chayan K. Nandi

**Affiliations:** School of Basic Sciences, Indian Institute of Technology Mandi, Kamand, Himachal Pradesh 175005 India

**Keywords:** Nanoparticle synthesis, Nanoparticles, Synthesis and processing

## Abstract

Insufficient purification and incomplete characterization pose a serious problem for attributing photoluminescence properties to carbogenic nanodots, especially those synthesized by bottom-up approaches. Here, we provide a roadmap for the successful future of these nanodots.

## Fallacies and artifacts in carbogenic nanodots

**Fig. 1:**
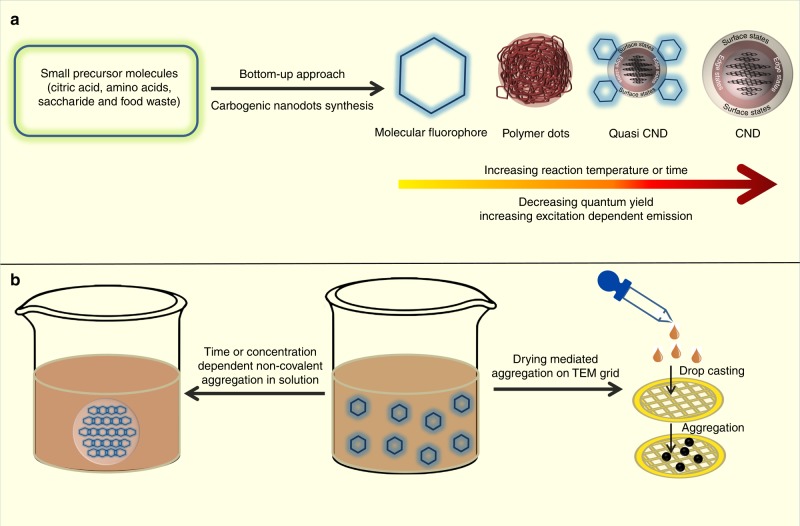
Bottom-up synthesis of carbogenic nanodots (CNDs) from small molecular precursors. **a** Synthesis may produce a complex mixture of molecular fluorophores, polymer dots, quasi CNDs, CNDs and **b** aggregated molecular fluorophores. The aggregated structure may also form by drying mediated aggregation of the fluorophore on the TEM grid

Carbogenic nanodots (CNDs), because of their superior optical properties, high photostability, quantum yield, aqueous solubility and low toxicity, have tremendous applications in optoelectronics, light harvesting, photovoltaics, drug delivery, bioimaging, catalysis and sensors. CNDs are very small in size (typically 2–5 nm) and show excitation dependent photoluminescence (i.e. the emission maximum peak shifts with the excitation wavelength). While the real chemical structure and the corresponding photoluminescence mechanism of these CNDs are still elusive, recent reports on bottom-up-synthesized CNDs pose another critical issue^1^. Recent research suggests that the fluorescence associated with CNDs may originate significantly from molecular fluorophores and/or their aggregates, quasi CNDs (molecular fluorophores attached to the core of CNDs) or polymer dots. These are produced as by-products (Fig. 1) or even as the sole product of CNDs synthesis. Moreover, the excitation dependent photoluminescence that is distinctive of CNDs is not observed in several cases^2^. As a result, the attribution of photoluminescence properties of the CNDs in several earlier reports may be affected by misleading artifacts and erroneous conclusions^3^.

For example, Shi et al. synthesized CNDs using citric acid and L-cysteine as the precursor molecules by a typical hydrothermal method^2^ and showed that an organic molecular fluorophore (with very small size and molecular weight) was actually responsible for the origin of fluorescence in the as-synthesized CNDs solution^4^. The above conclusion was drawn when they were able to synthesize the same fluorophore separately by reacting the same precursor molecules but in slightly milder experimental conditions. The fluorophore was purified and its chemical structure was confirmed by high resolution mass spectrometry (HRMS), nuclear magnetic resonance (NMR) and single crystal X-ray diffraction (XRD). Interestingly, all of the optical properties of this fluorophore, such as UV-VIS absorption, photoluminescence, quantum yield (QY) and fluorescence lifetime, were found to be similar to those of the as-synthesized CNDs solution. In addition, the excitation dependent photoluminescence typical of CNDs was neither present in the fluorophore nor in the CNDs solution. Finally, the authors confirmed the presence of large quantities of the fluorophore in the CNDs solution by dialyzing the solution using a 1000 Dalton molecular weight cut off filter and analyzing the optical properties of the dialysate and retentate. Interestingly, the dialysate, which contained the fluorophore, showed the same optical behavior as that of the as-synthesized CNDs solution, including UV-VIS absorption spectrum, high fluorescence QY, fluorescence excitation and emission spectra as well as excitation independent fluorescence. Meanwhile, the retentate, which contained the actual CNDs, displayed characteristic excitation dependent photoluminescence but with a very low QY (0.038%). The dominant peak in the HRMS spectrum was also attributed to the fluorophore. The authors concluded that the amount of real CNDs (with excitation dependent photoluminescence) produced in the synthesized solution was very small.

Song et al. identified a different molecular fluorophore in a CNDs solution^5^ synthesized by the hydrothermal method using citric acid and ethylenediamine as the precursor molecules. They proposed the formation of the fluorophore (with high QY and excitation independent photoluminescence) at a lower temperature, followed by the formation of polymer dots at a moderate temperature, and finally the formation of carbon cores (with very low QY and excitation dependent photoluminescence) at a higher temperature. They performed the dialysis and liquid chromatography-mass spectrometric (LC-MS) purification of the synthesized sample to confirm the presence of the small molecular fluorophore. The partial conversion from low molecular weight fluorophore to the π-conjugated carbon core with increasing temperature suggested that the unconverted fluorophore may bind to the larger π-conjugated domains to give rise to so-called quasi CNDs (CNDs coated with molecular fluorophores). This observation was supported by a few other recently studied CNDs systems^6–8^.

On a different note, several evidences suggest that presumed CNDs are actually aggregates of molecular fluorophores, either in ordered crystalline or amorphous form. For example, by performing HRMS analysis of the gel permeation chromatography-purified fluorophore that was obtained as a byproduct in CNDs synthesis in a hydrothermal method using citric acid and ethlylenetriamine, Zhang et al. concluded that the supramolecular aggregation of the fluorophores via hydrogen bonding showed excitation independent fluorescence with high QY^9^. The aggregated structure had the same topology and size as that of real reported CNDs. Reckmeier et al. showed that supposed CNDs synthesized by the hydrothermal treatment of ammonia and citric acid were actually the amorphous aggregates of the derivatives of citrazinic acid (a molecular fluorophore)^10^. A concentration-dependent aggregation of citrazinic acid was observed by ageing the solution at ambient conditions. With an increase in the concentration of citrazinic acid, the photoluminescence of the solution changed from excitation independent to excitation dependent. Interestingly the measured particle size (4.5 nm) of the aggregated structure using transmission electron microscopy (TEM) was found to be similar to that of real CNDs. On the other hand, Khan et al. reported the molecular fluorophore as a sole product during CNDs synthesis using only citric acid as precursor molecule in a hydrothermal synthesis^11^. The fluorophore underwent drying mediated hydrogen-bonded crystalline aggregation to show CND-like properties. They identified the fluorophore by solving the single crystal XRD and by measuring the diffusion coefficient using fluorescence correlation spectroscopy (FCS).

## Purifying the carbogenic nanodots

It is clear from the above reports that molecular fluorophores are often synthesized either as a sole product or in parallel to CNDs in the bottom-up hydrothermal synthesis. The synthesized molecular fluorophore, as an impurity, can contribute extensively to the fluorescence properties of the synthesized CNDs solution. As a result, it is extremely important to conduct a thorough purification and characterization to avoid any erroneous conclusions in CNDs research. The CNDs or quasi CNDs, considering their large mass difference from the small organic fluorophore molecule (<1 kDa for a typical fluorophore), can be separated easily using 1–3.5 kDa dialysis membrane. The similarity in the optical properties and the weight percentage of the dialysate (containing molecular fluorophore) with the retentate (containing CNDs) will provide insight into the contribution of the fluorophore to the optical and chemical properties of the as-synthesized CNDs^4,5^. Nevertheless, dialysis alone may not be a satisfactory method of purification, as it separates only a certain fraction of molecular weights that may contain different species within that cutoff range. Liquid chromatography purification, especially high performance liquid chromatography (HPLC) and gel permeation chromatography (GPC), which separate each component in a sample mixture based on polarity or size/molecular weight difference, will be extremely beneficial. As a result, the molecular fluorophore and its aggregated structure, polymer dots, and quasi CNDs can easily be separated from actual CNDs as ultrapure components.

## Guidelines to characterizing carbogenic nanodots

After complete separation of the individual species, the characterization of the proposed CNDs can be done by performing high resolution transmission electron microscopy (HR-TEM) and analysing its selected area electron diffraction (SAED) pattern, XRD, Raman spectroscopy and atomic force microscopy (AFM). The CNDs’ core, by its definition, can be both single crystalline multilayered graphitic carbon (with an interlayer spacing of ~0.34 nm for the 002-plane and 0.21 nm for the 001-plane) or it may consist of amorphous carbon^12–14^. Table 1 provides a set of comprehensive guidelines of the observed signatures for the identification of true CNDs. The above interlayer spacing can be obtained accurately by performing HR-TEM. In addition, the visible lattice fringes and the fast Fourier transform (FFT) honeycomb lattice structure or the hexagonal bright spots obtained in SAED will further confirm the crystalline nature. The sharp diffraction peak obtained in powder XRD at ~24˚ (Bragg’s angle, 2θ), corresponding to the spacing of 0.34 nm for 002-plane of the crystalline graphitic core, is another authentic confirmation. Considering the interlayer spacing of graphite (002-plane) of 0.34 nm, a 2–5 nm CNDs core will consist of about 6–14 layers of a single layer of graphene. The number of such layers can be quantitatively obtained by measuring the height profile using AFM^13^. Raman spectroscopy, a non-destructive versatile technique, has become the benchmark to identify the nature of graphene based materials^15^. Different forms of graphene have been identified by the well-known *D*, *G* and 2*D* bands. The *D* band (~1360 cm^−1^) characterizes the defect or the amorphous nature, the *G* band (~1580 cm^−1^) characterizes the in-plane vibration of the *sp*^2^ graphitic carbon, and the 2*D* band (~2680 cm^−1^) corresponds to the double resonance band. For a crystalline multilayered graphitic carbon core, in general one should not observe a *D* band; rather, an intense *G* band and a broad envelop of 2*D* band will be observed with the intensity ratio of the *G*/2*D* as 1:4^16^. Considering the edge defects created by the attachment of the functional groups, it is not surprising to observe a lesser intense *D* band (compared to *G* band). On the other hand, neither the interlayer spacing nor lattice fringes will be observed for an amorphous carbon core. In addition, a broad peak at Bragg’s angle (2θ) in XRD and a broad defective band in Raman spectroscopy will be observed for the amorphous CNDs core structure.

**Table 1:** Different characterization techniques for identifying pure crystalline CNDs

Techniques	Crystalline CNDs	Amorphous CNDs	Crystalline quasi CNDs	Aggregated fluorophores
HR-TEM	a. Size 2–5 nm, b. Lattice fringes c. Interlayer spacing of 0.34 nm for 002 plane d. Interlayer spacing of 0.21 nm for 001 plane	a. Size 2–5 nm, b. No lattice fringes c. No interlayer spacing	a. Size 2–5 nm, b. Lattice fringes c. Interlayer spacing of 0.34 nm for 002 plane d. Interlayer spacing of 0.21 nm for 001 plane	Almost similar to CNDs. A little variation may be observed based on the fluorophore structure
XRD	Sharp peak at ~24˚ for 002 plane	Broad peaks as compared to CND/Quasi CND	Sharp peak at ~24˚ for 002 plane	Sharp peak at ~24˚ for 002 plane
AFM	The height profile will confirm the size of 2–5 nm, topology and number of graphene layers in CNDs	The height profile will confirm the size of 2–5 nm and topology	The height profile will confirm the size of 3–5 nm, topology and number of graphene layers in CNDs	The height profile will confirm the size of 2–5 nm and topology
Raman Spectroscopy	a. Sharp G band at ~1580 cm^−1^, b. 2D band at ~2680 cm^−1^ c. less intense D band ~1360 cm^−1^ due to edge defect by surface functional groups	Predominant broad D bands or high D/G ratio	Same as CNDs as it contains the same crystalline core	Almost similar to CNDs. A little variation may be observed based on the fluorophore structure
TGA	No mass loss and mostly stable up to 800 °C	No mass loss and mostly stable up to 800 °C	Due to less thermal stability of molecular fluorophore extensive mass loss within 300 °C	Due to less thermal stability of molecular fluorophore extensive mass loss within 300 °C
Confocal Raman PL	Due to high thermal stability of carbon core no change in PL intensity at lower temperature ~300 °C	Due to high thermal stability of carbon core no change in PL intensity at lower temperature ~300 °C	Due to less thermal stability of molecular fluorophore huge reduction in PL intensity within 300 °C	Due to less thermal stability of molecular fluorophore complete reduction in PL intensity within 300 °C

It will be difficult to distinguish between CNDs and quasi CNDs by the above characterization as both of them contain similar carbon cores. However, the number of fluorophore units in quasi CNDs can be quantitatively obtained by thermogravimetric analysis (TGA), which works on the principle of mass loss with increasing temperature^17^. Considering the fact that the organic fluorophore will be volatile and have a much lower melting temperature than the graphitic core, the fluorophore will be destroyed (without disturbing the core) at its melting temperature and the total mass loss of the sample divided by the mass of the fluorophore will provide the number of fluorophores attached to the core in quasi CNDs. On the other hand, although there might be slight differences in either TEM or Raman spectroscopy data for aggregated molecular fluorophores, it will be difficult to distinguish them from CNDs. TGA again can be useful to differentiate between aggregated fluorophores and CNDs. It is obvious that the aggregated structure will have less thermal stability than the graphitic core in real CNDs and hence will show the mass loss at a lower temperature than CNDs core. X-ray photoelectron spectroscopy (XPS) can provide information about the surface functional groups and hence can distinguish the surface composition of CNDs, quasi CNDs and aggregated molecular fluorophores^18^. Finally, quantitative information about the contribution of the fluorophore in the optical properties in quasi CNDs can be obtained by in situ temperature dependent (following the same thermal stability of the fluorophore) photoluminescence measurements using confocal Raman spectroscopy^19^. The destruction of the fluorophore of the quasi CNDs will decrease the photoluminescence substantially. At a later stage, when all the fluorophore moieties are destroyed, the remaining photoluminescence should originate only from the core structure of the CNDs. On the other hand, complete quenching of fluorescence should be observed for the aggregated molecular fluorophore.

We hope that the above discussion will provide some guidance for the identification and characterization of CNDs and their associated photoluminescence properties in the bottom-up-synthesized complex products. It can be concluded that sufficient and adequate purification and characterization of the synthesized complex products are extremely crucial for the safe and successful future of the CNDs. While we discussed the key aspects of a few reported bottom-up hydrothermally synthesized CNDs, other hydrothermal or bottom-up syntheses using saccharides (fructose, glucose and chitosan), food waste, biomass, or other reagents still must be verified.
